# Alcohol-Induced Neuroleptic Malignant Syndrome Complicated by Severe Acute Rhabdomyolysis

**DOI:** 10.7759/cureus.81786

**Published:** 2025-04-06

**Authors:** Juan R Santos-Rivera, Regina J McPherson, Guillermo Izquierdo-Pretel

**Affiliations:** 1 Internal Medicine, Ponce Health Sciences University, Ponce, PRI; 2 Internal Medicine, Florida International University, Herbert Wertheim College of Medicine, Miami, USA; 3 Hospital Medicine, Jackson Memorial Hospital, Miami, USA

**Keywords:** alcohol-induced rhabdomyolysis, lithium, lithium-induced rhabdomyolysis, neuroleptic malignant syndrome, rhabdomyolysis

## Abstract

This case highlights a rare instance of alcohol-induced neuroleptic malignant syndrome (NMS) complicated by acute severe rhabdomyolysis, emphasizing the importance of recognizing atypical presentations of NMS. In June 2024, a 22-year-old male presented to the emergency department of a tertiary hospital in Florida with an acute alteration in mental status following alcohol consumption. Physical examination revealed neurological deficits alongside significant vital signs, including a temperature of 38°C, tachycardia, and hypertension. Key laboratory findings included a creatinine phosphokinase (CPK) level exceeding 100,000 U/L, aspartate aminotransferase (AST) above 3,000 U/L, alanine aminotransferase (ALT) above 500 U/L, and a lithium level below 0.20 mmol/L. The patient's medical history of bipolar disorder, managed with lithium, and recent alcohol intake suggest alcohol's role as a trigger for NMS in the context of lithium treatment, compounded by severe rhabdomyolysis. This case underscores the need for heightened clinical awareness of such complex interactions and highlights the critical importance of early recognition and multidisciplinary management to prevent potentially fatal complications.

## Introduction

We present the case of a neuroleptic malignant syndrome (NMS) induced by alcohol use and complicated by acute rhabdomyolysis. NMS is a rare but life-threatening condition commonly associated with the use of antipsychotic medications, particularly typical agents such as haloperidol and fluphenazine [1]. The reported incidence of NMS ranges from 0.01% to 3.2% in patients receiving neuroleptic therapy [2]. Although its pathophysiology is not fully understood, two primary mechanisms are proposed: dopamine D2 receptor antagonism, which disrupts hypothalamic thermoregulation and basal ganglia function, leading to hyperthermia and extrapyramidal symptoms, and a musculoskeletal fiber toxicity hypothesis, suggesting intracellular calcium dysregulation as the cause of muscle rigidity and hyperthermia. The latter is supported by the effectiveness of dantrolene, a muscle relaxant, in alleviating symptoms [3]. NMS can arise from dopamine receptor antagonists or abrupt withdrawal of dopaminergic agents [2]. Its clinical features include altered mental status, fever, muscle rigidity, and autonomic instability [1]. Prompt recognition is critical, as treatment involves discontinuing the offending agent and providing supportive care. For cases caused by abrupt withdrawal, reintroduction of the agent may be beneficial. Severe cases may require pharmacological interventions, such as bromocriptine (a dopamine agonist) or dantrolene [3]. NMS is identified clinically based on key features such as fever, muscle rigidity, and recent exposure to dopamine-blocking agents, along with at least two additional signs, including diaphoresis, dysphagia, tremor, incontinence, altered mental status, mutism, tachycardia, labile blood pressure, leukocytosis, or elevated creatinine phosphokinase (CPK) [2]. Initial evaluation of suspected NMS should include a complete metabolic panel, CPK level, urinalysis, and, if relevant, lithium levels. While the diagnosis is clinical, findings such as rhabdomyolysis, leukocytosis, and elevated transaminases support the diagnosis. With timely recognition and intervention, most patients recover within 2 to 14 days [1].

Rhabdomyolysis is a condition characterized by muscle injury that results in the release of muscle cell contents, including sarcoplasmic proteins and electrolytes, into the bloodstream [4]. Laboratory findings typically reveal elevations in myoglobin, CPK, lactate dehydrogenase (LDH), alanine aminotransferase (ALT), aspartate aminotransferase (AST), and electrolytes such as potassium [4]. Among these, elevated CPK levels are the most sensitive indicator of muscle injury [5]. However, an elevated CPK level does not necessarily correlate with the severity of muscle damage or the risk of renal injury [6]. There is no universally established serum cut-off for CPK elevation, though clinical practice often defines rhabdomyolysis as a CPK elevation of 5 to 10 times the upper limit of normal (100-400 IU/L) [7]. Several mechanisms can lead to muscle injury, including medications, crush injuries, seizures, alcohol or drug use, infections, prolonged immobility, and electrolyte imbalances [4]. However, not every muscle injury progresses to rhabdomyolysis. Its clinical consequences vary from mild CPK elevations to severe complications such as acute renal failure (ARF), cardiac arrhythmias, compartment syndrome, and disseminated intravascular coagulation (DIC) [8]. The most common symptoms are muscle weakness, myalgia, and dark red urine; however, this classic triad occurs in fewer than 10% of patients, and over half of the patients with rhabdomyolysis do not report muscle pain or weakness [5].

Despite diverse etiologies, the pathophysiology of rhabdomyolysis involves a common cascade. Sarcolemma damage - caused by direct trauma or metabolic stress - leads to adenosine triphosphate (ATP) depletion, increased intracellular calcium, and subsequent cell lysis. This process releases intracellular components into the bloodstream, contributing to systemic complications [9]. Early management focuses on aggressive hydration to prevent ARF. In severe cases, particularly when CPK levels exceed 30,000 IU/L, forced alkaline diuresis may be utilized in the absence of oliguria, anuria, or ARF [10].

Alcohol is a well-recognized trigger for acute rhabdomyolysis. Additionally, alcohol may interfere with the metabolism of antipsychotic medications, potentially leading to elevated drug levels and triggering NMS [11].

NMS can directly lead to rhabdomyolysis due to sustained muscle rigidity and hyperthermia, which result in muscle cell breakdown and the release of intracellular contents. This complication, while not present in all cases of NMS, can significantly worsen prognosis if unrecognized. Rhabdomyolysis in the setting of NMS is thought to arise from the same pathophysiologic mechanism responsible for rigidity - namely, dopaminergic disruption and possible calcium dysregulation within muscle fibers.

## Case presentation

A 22-year-old male with a history of bipolar disorder, managed with lithium, olanzapine, and aripiprazole, was brought to the emergency department (ED) as a stroke alert due to acute altered mental status. He had been on a cruise and reportedly consumed alcohol the evening prior. According to his proxy, he felt febrile after drinking and was found the next morning confused and minimally responsive. His last known well time was approximately seven hours before arrival. There was no confirmed use of illicit substances or supplements.

In the ED, his National Institutes of Health Stroke Scale (NIHSS) score was 11. Vital signs included a temperature of 38°C, heart rate of 112 bpm, blood pressure of 145/79 mmHg, respiratory rate of 20, oxygen saturation of 94%, and glucose level of 145 mg/dL. Neurologic examination revealed somnolence, sluggish bilateral pupils, minimal withdrawal to painful stimuli, and decreased strength: 3/5 in the upper extremities and right lower extremity, with minimal movement in the left lower extremity.

Initial laboratory evaluation revealed leukocytosis, markedly elevated liver transaminases (AST > 3,000 U/L, ALT > 500 U/L), and extreme CPK elevation >112,000 U/L. His lithium level was subtherapeutic (<0.20 mmol/L). Electrolytes and renal function were within normal limits. Urinalysis revealed brown, turbid urine that was heme-positive without red blood cells, consistent with myoglobinuria and rhabdomyolysis. Urine toxicology was negative. Full laboratory results from admission and discharge are summarized in Table 1.

**Table 1:** Laboratory results on admission and at discharge BUN: blood urea nitrogen; AST: aspartate aminotransferase; ALT: alanine aminotransferase; CPK: creatinine phosphokinase; WBC: white blood cell count

Laboratory values	Admission time	Discharge time	Reference ranges
Sodium	143 mmol/L	138 mmol/L	137-145 mmol/L
Potassium	4.9 mmol/L	4.5 mmol/L	3.6-5.0 mmol/L
Creatinine	1.20 mg/dL	0.9 mg/dL	0.66-1.25 mg/dL
Blood urea nitrogen (BUN)	23 mg/dL	10 mg/dL	9.0-20 mg/dL
AST	3047 unit/L	1775 unit/L	15-46 unit/L
ALT	555 unit/L	667 unit/L	9.0-45 unit/L
Total bilirubin	1.5 mg/dL	0.6 mg/dL	0.2-1.3 mg/dL
CPK	112,691 unit/L	23,091 unit/L	57-374 unit/L
WBC	13.9 x 10^3^/mcL	6.2 x 10^3^/mcL	4.0-10.5 x 10^3^/mcL
Hemoglobin	18.3 g/dL	16.8 g/dL	13.3-16.3 g/dL
Hematocrit	51.8%	50.0%	39.0-47.1%
Platelet	230 x 10^3^/mcL	222 x 10^3^/mcL	140-400 x 10^3^/mcL
Lithium level	<0.20 mmol/L	<0.20 mmol/L	0.50-1.20 mmol/L

**Figure 1:**
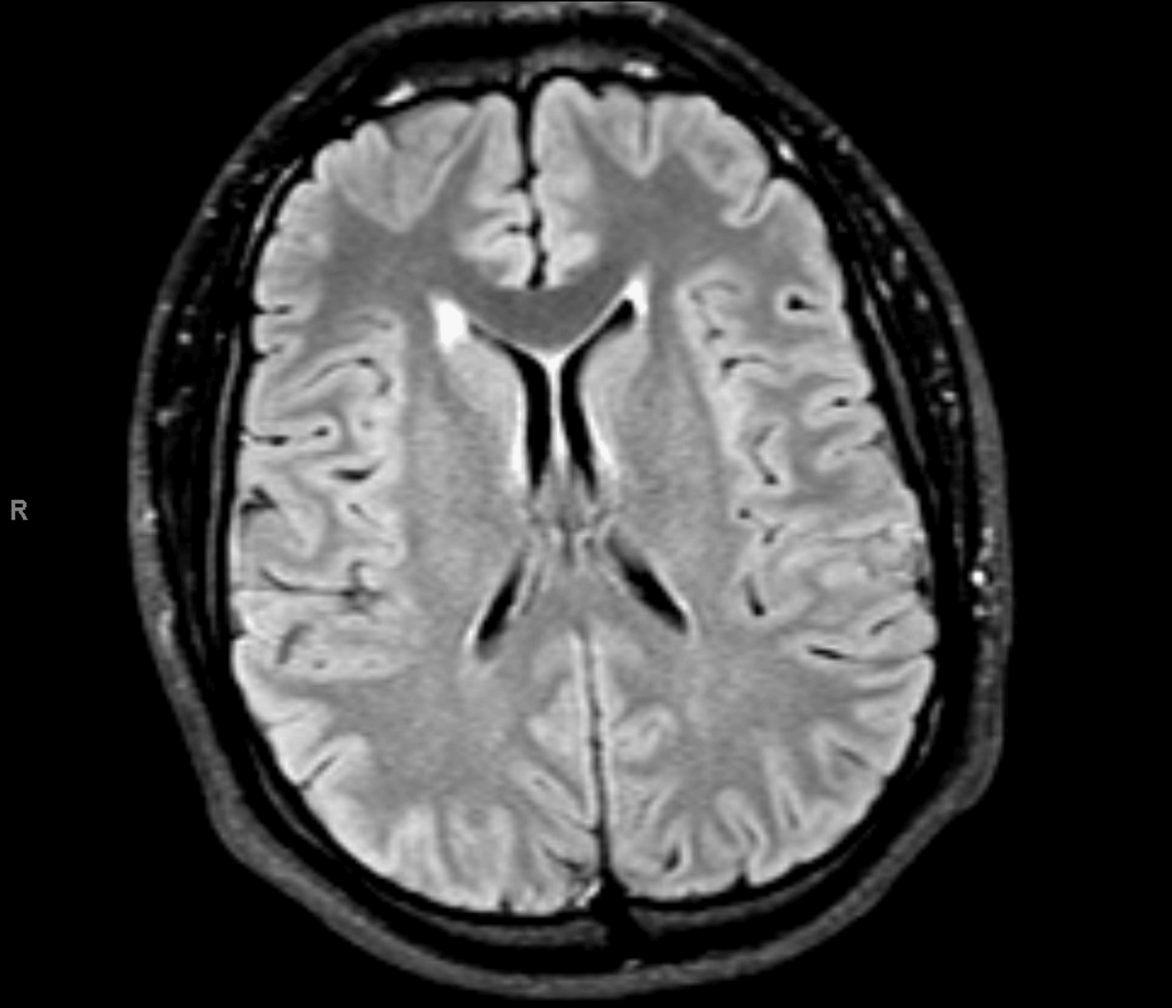
Brain MRI Imaging in a 22-year-old male with acute altered mental status and suspected NMS showed no evidence of acute ischemia, hemorrhage, mass lesions, or abnormal contrast enhancement. These findings reinforced the diagnosis of NMS by ruling out structural or vascular causes of his presentation and correlating with clinical signs of rhabdomyolysis and systemic inflammation. NMS: neuroleptic malignant syndrome

A brain MRI showed no evidence of acute ischemia, hemorrhage, mass lesions, or abnormal enhancement, effectively ruling out structural causes for his symptoms (Figure 1).

The patient met criteria for systemic inflammatory response syndrome (SIRS). Broad-spectrum antibiotics were initiated while awaiting blood and urine cultures and chest imaging. Neurology was consulted, and all antipsychotic medications were discontinued due to concern for NMS. He was admitted to the medical intensive care unit (MICU) and treated with intravenous fluids and supportive care.

Within 24 hours, his mental status returned to baseline, and vital signs normalized. Antibiotics were discontinued after cultures returned negative. His CPK trended down with continued hydration. By day 5, it had decreased from 112,691 U/L to 23,091 U/L. Despite the persistently elevated CPK, the patient requested discharge against medical advice, citing plans to return to California. He was counseled extensively and provided with detailed instructions for outpatient psychiatric follow-up and return precautions.

## Discussion

This case illustrates a rare presentation of alcohol-induced NMS complicated by acute severe rhabdomyolysis. The diagnosis of NMS was substantiated by the presence of key clinical features, including fever, muscle rigidity, leukocytosis, elevated transaminases, altered mental status, tachycardia, and markedly elevated CPK levels (112,691 U/L on admission, 23,091 U/L at discharge) [3,7]. The patient’s history of antipsychotic use, excessive alcohol consumption, and rapid clinical improvement following cessation of antipsychotics underscores the role of these triggers in the development of NMS and rhabdomyolysis.

Rhabdomyolysis in this patient was evident from the extreme elevation in CPK levels (112,691 U/L on admission), with associated transaminase elevations and SIRS criteria [3]. While NMS can independently cause rhabdomyolysis due to sustained muscle rigidity and hyperthermia, alcohol may have exacerbated the condition through mechanisms such as direct muscle toxicity, metabolic stress, or immobility [11]. This dual contribution likely amplified the severity of the rhabdomyolysis.

The primary concern in managing rhabdomyolysis is preventing acute kidney injury (AKI), which may result from myoglobinuria and renal hypoperfusion. Myoglobinuria, a nephrotoxic consequence of rhabdomyolysis, is particularly concerning in cases of severe muscle injury [6]. Additionally, systemic inflammatory responses and hypovolemia associated with rhabdomyolysis further predispose patients to AKI [3]. Studies suggest that bicarbonate therapy and mannitol may be beneficial in preventing renal complications by alkalinizing the urine and promoting diuresis, although evidence remains inconclusive [12]. In this case, aggressive intravenous hydration effectively mitigated these risks. The rapid downtrend of CPK levels (to 23,091 U/L at discharge) and absence of AKI underscore the importance of early recognition and intervention in rhabdomyolysis management.

Alcohol’s potential role as a trigger for NMS has been noted but remains poorly understood. Acute alcohol intoxication may interfere with the metabolism of psychotropic medications, such as antipsychotics, leading to increased drug levels and enhanced dopamine receptor blockade [11]. This interaction may have contributed to the development of NMS in this patient. The interplay between alcohol, antipsychotic medications, and muscle toxicity warrants further exploration, particularly in individuals with predisposing factors such as bipolar disorder.

Emerging evidence from pharmacovigilance analyses further supports the complexity of NMS pathogenesis. A recent signal detection study using the Japanese Adverse Drug Event Report (JADER) database identified not only antipsychotics but also anti-Parkinsonian, anti-anxiety, and antiepileptic drugs as potential contributors to NMS [13]. These agents appear to act primarily through neuroactive ligand-receptor interactions and disruptions in dopaminergic and serotonergic synapses. Additionally, several drug-drug interactions (DDIs) were found to significantly increase NMS risk, including combinations of atypical antipsychotics with lithium. This is particularly relevant given evidence that chronic lithium exposure may limit dopamine signaling, potentially enhancing the D2 receptor blockade of agents like risperidone or olanzapine. These findings reinforce the importance of monitoring for NMS in patients prescribed multiple psychotropic medications, even when drug levels are subtherapeutic or when non-antipsychotic agents are involved. Notably, while the JADER analysis did not include alcohol as a variable, its absence highlights a critical limitation in spontaneous reporting databases - namely, the underrepresentation of lifestyle or substance-related factors, which may act as unmeasured contributors to drug interactions or toxicity. In this case, alcohol’s role in impairing hepatic metabolism and potentially amplifying psychotropic effects cannot be overlooked.

This case highlights the complexity of diagnosing and managing NMS, particularly in atypical presentations. Prompt discontinuation of the offending agents and initiation of supportive care were critical to achieving a favorable outcome in this case. The absence of AKI and the rapid clinical recovery emphasize the importance of timely intervention and comprehensive monitoring during hospitalization.

From a broader perspective, this case underscores the need for increased awareness among healthcare professionals about the potential interactions between alcohol and psychotropic medications. Educating patients on the risks associated with alcohol consumption while on antipsychotics is equally important. Such education can help prevent life-threatening complications like NMS and rhabdomyolysis [11].

Finally, this case highlights the importance of further research into the mechanisms by which alcohol interacts with antipsychotic medications and its role in triggering severe syndromes like NMS. Understanding these mechanisms more thoroughly could lead to improved prevention strategies and tailored interventions for at-risk populations.

## Conclusions

NMS and acute rhabdomyolysis are rare but serious complications that may follow excessive alcohol consumption, particularly in individuals on antipsychotic medications. Alcohol can interfere with the metabolism of these medications, potentially triggering NMS. Early recognition is crucial to promptly discontinue the offending agent and provide supportive care, which can significantly improve outcomes. In parallel, acute rhabdomyolysis should be recognized as a medical emergency in its own right, especially in the setting of elevated CPK levels and heme-positive urine without red blood cells. Prompt and aggressive intravenous hydration is critical to reduce the risk of AKI and other systemic complications. Serial CPK monitoring and renal function assessment are essential components of management.

The diagnosis of NMS is challenging due to its variable presentation, requiring healthcare professionals to be well-versed in the diagnostic criteria and diligent in obtaining a thorough patient history to identify potential triggers. Counseling patients on the risks of alcohol use in conjunction with antipsychotics and ensuring close follow-up after hospital discharge are essential steps to prevent recurrence and promote long-term well-being.
